# Molecular Basis of Plant Profilins’ Cross-Reactivity

**DOI:** 10.3390/biom13040608

**Published:** 2023-03-28

**Authors:** María G. Terán, Benjamín García-Ramírez, Israel Mares-Mejía, Enrique Ortega, Andrea O’Malley, Maksymilian Chruszcz, Adela Rodríguez-Romero

**Affiliations:** 1 Instituto de Química, Universidad Nacional Autónoma de México, Mexico City 04510, Mexico; magateol@gmail.com (M.G.T.); benjamin.garcia.ramirez@live.com (B.G.-R.); mares27@hotmail.com (I.M.-M.); 2 Instituto de Investigaciones Biomédicas, Universidad Nacional Autónoma de México, Circuito Exterior, Ciudad. Universitaria, Coyoacán, Mexico City 04510, Mexico; ortsoto@iibiomedicas.unam.mx; 3 Department of Chemistry and Biochemistry, University of South Carolina, Columbia, SC 29209, USA; aomalley@email.sc.edu (A.O.); chruszcz@msu.edu (M.C.); 4 Department of Biochemistry and Molecular Biology, Michigan State University, East Lansing, MI 48824, USA

**Keywords:** monoclonal antibodies, cross-reactivity, plant profilins, identification, conformational epitopes

## Abstract

Profilins are ubiquitous allergens with conserved structural elements. Exposure to profilins from different sources leads to IgE-cross-reactivity and the pollen–latex–food syndrome. Monoclonal antibodies (mAbs) that cross-react with plant profilins and block IgE-profilin interactions are relevant for diagnosis, epitope mapping, and specific immunotherapy. We generated IgGs mAbs, 1B4, and 2D10, against latex profilin (anti-rHev b 8) that inhibit the interaction of IgE and IgG4 antibodies from sera of latex- and maize-allergic patients by 90% and 40%, respectively. In this study, we evaluated 1B4 and 2D10 recognition towards different plant profilins, and mAbs recognition of rZea m 12 mutants by ELISAs. Interestingly, 2D10 highly recognized rArt v 4.0101 and rAmb a 8.0101, and to a lesser extent rBet v 2.0101, and rFra e 2.2, while 1B4 showed recognition for rPhl p 12.0101 and rAmb a 8.0101. We demonstrated that residue D130 at the α-helix 3 in profilins, which is part of the Hev b 8 IgE epitope, is essential for the 2D10 recognition. The structural analysis suggests that the profilins containing E130 (rPhl p 12.0101, rFra e 2.2, and rZea m 12.0105) show less binding with 2D10. The distribution of negative charges on the profilins’ surfaces at the α-helices 1 and 3 is relevant for the 2D10 recognition, and that may be relevant to explain profilins’ IgE cross-reactivity.

## 1. Introduction

Pollen profilins are an important cause of respiratory allergies, such as rhinoconjunctivitis and asthma. In Europe, 20–30% of allergic individuals to pollen are sensitized to profilins [1,2]. Profilins are panallergens due to their ubiquity in eukaryotic cells, high identity sequence, and similar folding of these proteins from different sources [3]. It has also been reported that respiratory allergic patients to pollen profilins may develop another allergic response, including oral allergic syndrome and anaphylaxis [4,5]. These highlight the importance of plant profilin sensitization in allergic diseases [6].

Monoclonal antibodies (mAbs) against specific allergens have become effective biological tools for diagnosis and IgE-epitope mapping. Moreover, they have emerged with potential clinical applications to treat allergies [7,8]. To date, mAbs against profilins have been used for their quantification from natural sources [9,10] and mapping antigenic determinants through synthetic overlapping peptides [11]. Recently, we reported structural studies of Fab 2F5/IgE in complex with recombinant latex profilin (rHev b 8) (PDB 7SBD). We identified the relevant interactions that stabilize the complex and the Hev b 8 conformational epitopes at α-helices 1 and 3, including residue N98 from the loop that connects β-strands 6 and 7. One of these interactions involved residue D128 in the epitope, which establishes two salt bridges with R50 and R52 (CDR2-H) of the paratope, and another in which residue N98 interacts through hydrogen bonds with T55 in the CDR2-H. To determine if these residues are involved in IgE cross-reactivity, we used maize profilin (Zea m 12), which superimposes well with Hev b 8 (RMSD: 0.3 Å) compared to other profilins; however, the mAb 2F5/IgE did not recognize it. A superposition of rZea m 12 on the Fab 2F5/IgE-rHev b 8 structure indicated differences in these amino acids: Hev b 8 has N98 and D128, while rZea m 12 has G98 and E128. Therefore, we generated two rZea m 12 mutants: E128D and the double mutant G98N-E128D, and evaluated the recognition with mAb 2F5/IgE, which resulted in 16% with the single rZea m 12 mutant, while the double mutant showed 56% IgE recognition, compared to rHev b 8 recognition [12].

We have also produced murine IgG monoclonal antibodies (mAbs) 1B4 and 2D10 against latex profilin (anti-rHev b 8). To define rHev b 8 epitopes recognized by these IgG mAbs and compare them with those identified by the 2F5/IgE, we performed molecular modeling of the Fv regions of both mAbs and docking simulations of the Fvs-rHev b 8 complexes. According to these analyses, the 2D10 epitope comprises α-helices 1 and 3 at the amino and carboxy-terminal regions, such as the region recognized by 2F5/IgE. In contrast, 1B4 recognized the opposite side on the profilin surface [13]. These two mAbs inhibited polyclonal antibodies (from latex allergic patients’ sera), 23% when we used 1B4 and 74% when we used 2D10. In addition, when we used a mixture of these two mAbs, they could mimic the interaction of IgE and IgG4 antibodies from the sera of latex and maize-allergic patients, inhibiting their binding to profilin by 90% and 40%, respectively [13]. Interestingly, when we used sensitized rat basophilic leukemia cells with the 2F5/IgE antibody and formed a complex of rHev b 8 with 2D10 (rHev b 8-2D10), degranulation was prevented, corroborating that 2D10 blocks the interaction of 2F5/IgE with this latex allergen [13].

The mAbs 1B4 and 2D10 have only been studied with maize and latex profilins. Their recognition with other plant profilins has not been evaluated nor analyzed at the structural level, which may explain the bases of the cross-reactivity of profilins. Therefore, in this work, we aimed to assess mAbs 1B4 and 2D10 recognition towards allergenic plant profilins, focusing on profilins from trees (rBet v 2.0101/birch, rFra e 2.2/ash), weeds (rAmb a 8.0101/ragweed, and rArt v 4.0101/mugwort), grass (rPhl p 12.0101/Timothy grass), and food (Zea m 12.0105/maize). Additionally, we used rZea m 12.0105 mutants to characterize the recognition sites of the two mAbs. Critical amino acids involved in mAbs recognition were detected through sequence alignment and comparison with the rHev b 8 epitopes reported from the structure of the complex Fab 2F5/IgE-rHev b 8 (PDB 7SBD). Specific mAbs showing high recognition for diverse profilins may represent a promising tool in the allergy field for diagnosis, immuno-localization studies, and even the development of personalized immunotherapy.

## 2. Materials and Methods

### 2.1. Purification of nFra e 2.2 from Ash Pollen

Inflorescences were collected from *Fraxinus excelsior* trees in Mexico City. The pollen grains were washed with ethanol-acetone 1:1 (*v/v*) and centrifuged (8000× *g*, 15 min). The pellet was resuspended in 50 mM sodium carbonate-bicarbonate buffer, pH 8, with 5 mM EDTA and 1 mM PMSF, and the solution was mixed for two hours and centrifuged (8000× *g*, 30 min). Ash profilin was purified by affinity chromatography using a poly-L-proline resin in batch. The protein extract was incubated overnight at 4 °C and washed with equilibration buffer (50 mM sodium carbonate-bicarbonate). Profilin was eluted with 4 M guanidinium chloride, dialyzed against 50 mM Tris-HCl buffer pH 8, and stored at 4 °C overnight. The presence and purity of the proteins were verified by SDS-PAGE 15%. The profilin bands were cut and sent to Arizona State University for sequencing.

### 2.2. Ash Profilin (rFra e 2) Expression and Purification

The identified peptides from pollen belonged to isoform Fra e 2.2, reported previously [14] Therefore, the gene that encodes this protein, deposited in the NCBI database (GenBank: AF526295.1), was synthesized in the pET-28c (+) vector by GenScript. The pET28c-rFra e 2.2 vector was cloned for the recombinant allergen expression in *E. coli* Rosetta (DE3) cells. Cells were grown in Luria broth (LB) medium supplemented with 30 μg/mL kanamycin at 37 °C. Protein expression was induced with 0.5 mM isopropyl-thiol-β-D-1-galactopyranoside when it reached an optical density of 0.6 at 600 nm, and incubation was continued for 6 h at 30 °C. Cells were harvested by centrifugation (15,300× *g*, 4 °C, 20 min) and resuspended in a lysis buffer (50 mM Tris pH 8, 300 mM NaCl, 20 mM imidazole, 1 mM PMSF), sonicated (60 cycles in 10 s intervals, with a stop time of 30 s to complete 10 min, 4 °C) and centrifuged (26,000× *g*, 4 °C, 30 min). The supernatant was filtered and loaded onto a 5 mL Ni-NTA affinity column, equilibrated with 50 mM Tris-HCl pH 8, 300 mM NaCl, 20 mM imidazole, and washed with the same buffer. Finally, the protein was purified with anion exchange chromatography with a Mono Q HR 5/5 column in an ÄKTA FPLC (Amersham Biosciences, Uppsala, Sweden), washed with 40 mM NaCl, and eluted with 160 mM NaCl. The Histag was cleaved overnight at 37 °C for 2 h using a recombinant tobacco virus protease (TEV) in 50 mM Tris-HCl pH 8, 0.5 mM EDTA, and 5 mM DTT buffer. Then, it was dialyzed against 100 mM Tris-HCl pH 8.6 and stored at −20 °C. Finally, Fra e 2.2 was loaded in an SDS-PAGE 12%. We determined the molecular mass by matrix-assisted laser desorption/ionization-time of flight (MALDI-TOF) mass spectrometry (Microflex; Bruker Scientific LLC, Billerica, MA, USA). We used 10 mg of the protein for the analysis and thaumatin (22 kDa) and hevein (4.7 kDa) as calibration standards. The matrix used was a saturated solution of sinapinic acid in 30% (*v/v*) aqueous acetonitrile and 0.1% (*v/v*) trifluoroacetic acid. The results were analyzed using Bruker’s Flex analysis 3.0 TM software.

### 2.3. Recombinant Profilin Allergens Art v 4, Amb a 8, Phl p 12 and Bet v 2

The recombinant profilin allergens Art v 4.0101—*Artemisa vulgaris* (mugwort) (Uniprot identifier-Q8H2C9), Amb a 8.0101—*Ambrosia artemisiifolia* (ragweed) (Q2KN24), Phl p 12.0101—*Phleum pratense* (Timothy grass) (P35079), and Bet v 2.0101—*Betula verrucosa* (birch) (P25816) were produced using previously described protocols [15,16]. Purified profilins (1 mg/mL) were dialyzed against PBS buffer and stored at −80 °C.

### 2.4. mAbs (Anti-rHev b 8) Purification

Murine mAbs IgG 1B4 and IgG 2D10 were produced by hybridoma cells, as previously described [13]. Briefly, these cells were grown in RPMI-1640 medium with sodium pyruvate, L-glutamine, non-essential amino acids, antibiotics, and 3% fetal bovine serum (FBS) incubated at 37 °C with 5% CO_2_. The culture supernatant was centrifuged (2000× *g* for 20 min) and filtered through a 0.22 µm membrane. Antibodies 1B4 and 2D10 were purified by affinity chromatography with an Affi-Gel-resin (Biorad, Berkeley, CA, USA) covalently bound to rZea m 12, previously equilibrated with PBS buffer. The antibody was eluted with 0.2 M Glycine-HCl buffer pH 2.8 and 1 M NaCl buffer, and the collected fractions (800 µL) were received in 200 µL 2 M Tris HCl buffer pH 8. The purity of the antibodies was analyzed by 10% SDS-PAGE. The purified antibodies were dialyzed against PBS, concentrated to 1 mg/mL, and stored at −20 °C.

### 2.5. Recognition of Plants Profilins (rArt v 4, rAmb a 8, rPhl p 12, rBet v 2, and rFra e 2) by mAbs 2D10 and 1B4 (Anti-rHev b 8)

We performed indirect ELISA experiments by coating microplates with 100 µL of a 10 µg/mL solution of rHev b 8, rZea m 12, rFra e 2, rArt v 4, rAmb a 8, rPhl p 12, and rBet v 2 at 37 °C using PBS, pH 7.4, incubated at 4 °C overnight. Previously, we determined the optimum mAb 2D10 concentration for recognition of rHev b 8, to be 2 µg/mL. We used albumin (BSA), glutathione S-transferase from *Taenia solium*, and Hev b 7 (patatin from *H. brasiliensis*) as negative controls. Between steps, wells were washed with 200 µL of 0.05% Tween 20 in PBS. The microplate was blocked with 150 µL, 1% BSA in PBS for two hours at 37 °C. Antibodies 1B4 and 2D10 were incubated at 2 µg/mL, diluted in PBS, and incubated at 37 °C for 2 h. The plate was then washed, and the secondary antibody, a goat anti-mouse IgG (Pierce Thermo-Scientific, Rockford, IL, USA, 31430) HRP-labeled (Pierce Thermo-Scientific, 31430), was added per well and set for 30 min at 37 °C. ABTS substrate (Life Technologies, 00-2024) was added and incubated for 30 min. Plates were read at 405 nm using a Cytation 3 plate reader (BioTek Instruments Inc., Winooski, VT, USA), and the absorbance of the negative control wells was subtracted from each value. Each condition was assayed in triplicate. The reported absorbance values and the corresponding standard deviation were averaged from three independent experiments for each condition.

### 2.6. Characterization of Recognition Sites of mAbs (1B4 and 2D10) with rZea m 12 Mutants

This study used the rZea m 12 mutants to characterize mAb 1B4 and 2D10 recognition sites by indirect ELISA experiments with rHev b 8 and rZea m 12 as controls. The microplates were coated with 100 µL of a 10 µg/mL solution of rHev b 8, of rZea m 12 E128D single mutant, or of rZea m 12 G98N-E128D double mutant. The following steps were the same as described above. Plates were read at 405 nm using a Cytation 3 plate reader (BioTek Instruments Inc., Winooski, VT, USA), and the absorbance of the negative control wells was subtracted from each value. Each condition was assayed in triplicate. The reported absorbance value and the corresponding standard deviation was averaged from three independent experiments.

### 2.7. Evaluation of the Recognition of Sera from Maize and Latex Allergic Patients to rZea m 12 Mutants

We performed indirect ELISA experiments to identify the relevant residues involved in IgE binding with sera of patients allergic to maize and latex profilins. Human sera from controls and profilin-allergic individuals with medical records of allergies to latex and maize were volunteers. We used a group of five individuals with an allergy to rHev b 8 or rZea m 12 (positive skin prick tests, allergic rhinitis symptoms, and food allergy syndrome). We evaluated the IgE-specificity by ELISA to rZea m 12 and rHev b 8 of the serum of maize- and latex-allergic patients and negative controls.

The microplate was coated with 100 µL of a 10 µg/mL solution of rHev b 8, rZea m 12, and rZea m 12 mutants. All the following incubation steps were performed at 4 °C overnight. Between steps, wells were washed with 200 µL of 0.05% Tween 20 in PBS. The microplate was blocked with 150 µL of 1% BSA in PBS. The specific-IgE binding to rHev b 8, rZea m 12, and rZea m 12 mutants was evaluated with a pool of sera from five allergic individuals that tested positive for maize. The latter and the control (two negative sera) were prepared 1:5 in PBS. The microplate was then washed, and the secondary antibody (anti-human IgE HRP-labeled, ε-chain specific; Sigma Aldrich, St. Louis, MI, USA; A9667) was added per well and incubated for 2 h at 37 °C. The following steps were the same as described for indirect ELISA. Each condition was assayed in triplicate. The reported absorbance value and the corresponding standard deviation was averaged from three independent experiments.

### 2.8. Computational Analyses

The sequences of profilins Amb a 8.0101, Art v 4.0101, Bet v 2.0101, Phl p 12.0101, Zea m 12.0105, Hev b 8.0102, and Fra e 2.2 sequences were aligned in the Clustal Omega server https://www.ebi.ac.uk/Tools/msa/clustalo/ (accessed on 23 November 2022). The Alpha Fold server [17] was used to obtain the Fra e 2.2 model. Alignment of profilins’ structures Hev b 8 (PDB 5FDS), Art v 4 (PDB 5EM0), Amb a 8 (PDB 5EM1), Phl p 12 (PDB 7KYW), and Bet v 2 (PDB 5NZB) was performed with PYMOL (DeLano, 2002 The PyMOL Molecular Graphics System, Version 2.0; Schrödinger, LLC, New York, NY, USA), and its electrostatic potential surfaces were obtained with APBS [18].

### 2.9. Statistical Analyses

The statistical analysis of the ELISAs was performed using the one way-analysis of variance (ANOVA), followed by an all-pairwise multiple comparison procedure (Šídák method). A *p*-value < 0.05 was considered statistically significant [19]. All summary statistics and analyses were performed using the Prism 8.0 software (GraphPad Software, La Jolla, CA, USA) https://www.graphpad.com (accessed on 23 November 2022).

## 3. Results

### 3.1. Evaluation of mAbs 2D10 and 1B4 (Anti-rHev b 8) Recognition towards Allergenic Profilins from Plants

**Figure 1:**
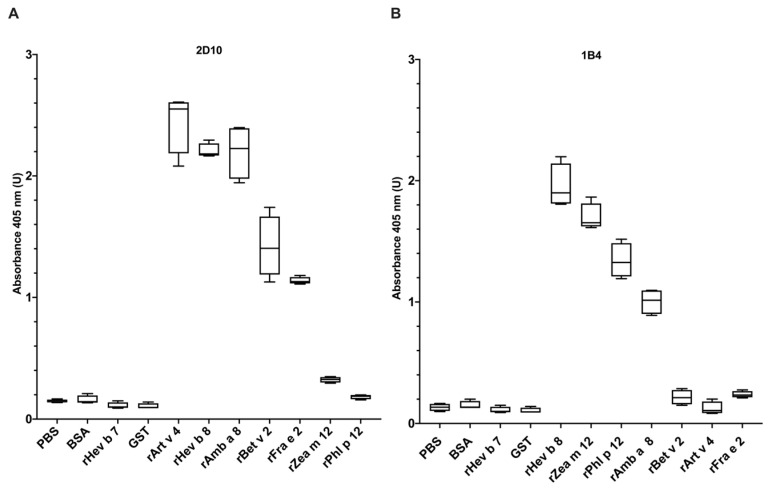
mAbs 2D10 and 1B4 (anti-rHev b 8) binding toward recombinant allergenic profilins rArt v 4, rAmb a 8, rBet v 2, rPhl p 12, rFra e 2, rZea m 12 and rHev b 8. The box and whisker plot shows the binding measured by indirect ELISA and represents the mean and the standard deviation of three experiments. (**A**) mAb 2D10 shows binding with rArt v 4, rHev b 8, rAmb a 8, rBet v 2, and rFra e 2. (**B**) mAb 1B4 shows binding with rHev b 8, rZea m 12, rPhl p 12, and rAmb a 8. The negative controls are BSA, rHev b 7 (patatin), glutathione S-transferase (GST) from *Tenia solium* and PBS as background control, and the positive control is rHev b 8.

To determine 2D10 and 1B4 mAbs recognition towards the profilins rArt v 4, rAmb a 8, rBet v 2, rPhl p 12, rFra e 2, rZea m 12, and rHev b 8, we performed ELISA experiments (Figure 1A). Remarkably, mAb 2D10 strongly recognized Art v 4 compared to rHev b 8, whereas rAmb a 8 binding was 88%, considering Art v 4 recognition as 100%. rBet v 2 exhibited a binding of 54% and rFra e 2 of 42%; the lowest recognition was for rZea m 12 with 11%, and no recognition for rPhl p 12 (Figure 1A). For mAb 1B4, and considering its binding with Hev b 8 as 100%, Zea m 12 binding was 89%, Phl p 12 was 72%, and with rAmb a 8 was 56%. rArt v 4, rBet v 2, and rFra e 2 were not recognized by this mAb, as shown in Figure 1B. These data suggest that mAb 2D10 and 1B4 show different recognition patterns on plant profilins.

### 3.2. Characterization of Recognition Sites of IgG mAbs 1B4 and 2D10 (anti-rHev b 8) to the Two Maize Profilin Mutants (rZea m 12)

**Figure 2:**
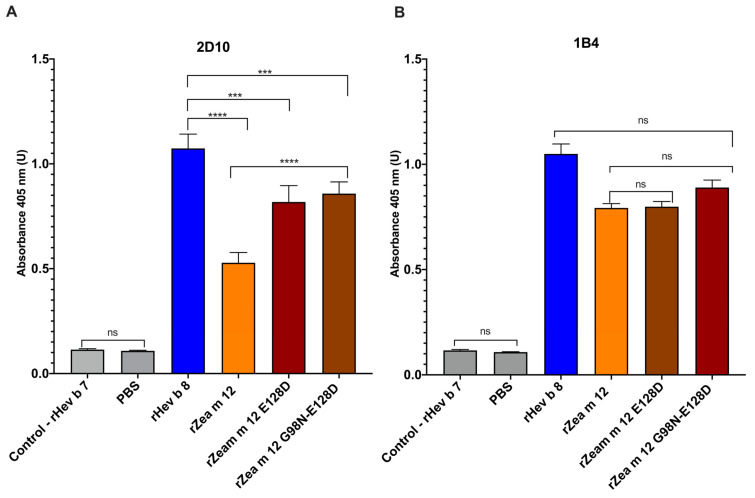
Characterization of recognition sites of mAbs (1B4 and 2D10) with Zea m 12 mutants. The bars show the recognition of rHev b 8, rZea m 12, the single mutant rZea m 12-E128D, and the double mutant rZea m 12 G98N-E128D measured by indirect ELISA. (**A**) mAb 2D10 strongly binds rZea m 12-E128D. (**B**) mAb 1B4 shows no significant difference in binding with rZea m 12 and its mutants. The negative controls are BSA and PBS as background control, and the positive control is rHev b 8. The standard deviation of three different experiments is shown. The statistical analysis was performed by Sidak’s multiple comparisons tests (ns, not significant, all *p* < 0.001 are summarized with three asterisks, and *p* < 0.0001 with four asterisks).

To identify the residues on the two maize profilin mutants (rZea m 12) recognized by mAbs 2D10 and 1B4, we performed an indirect ELISA. Figure 2A shows an increased binding of 2D10 to the mutants rZea m 12 E128D and rZea m 12 G98N-E128D, compared to rZea m 12. These results confirm that D128 is an essential residue that interacts with 2D10. On the other hand, 1B4 did not show critical recognition changes with these two maize profilin mutants (Figure 2B), suggesting that 1B4 does not recognize residues in amino and carboxyl-terminal α-helices 1 and 3 in profilins and does not share the binding site with 2D10.

### 3.3. Increased Binding of IgE from a Pooled Sera from Maize and Latex Allergic Patients to the Double Mutant rZea m 12 G98N-E128D

**Figure 3:**
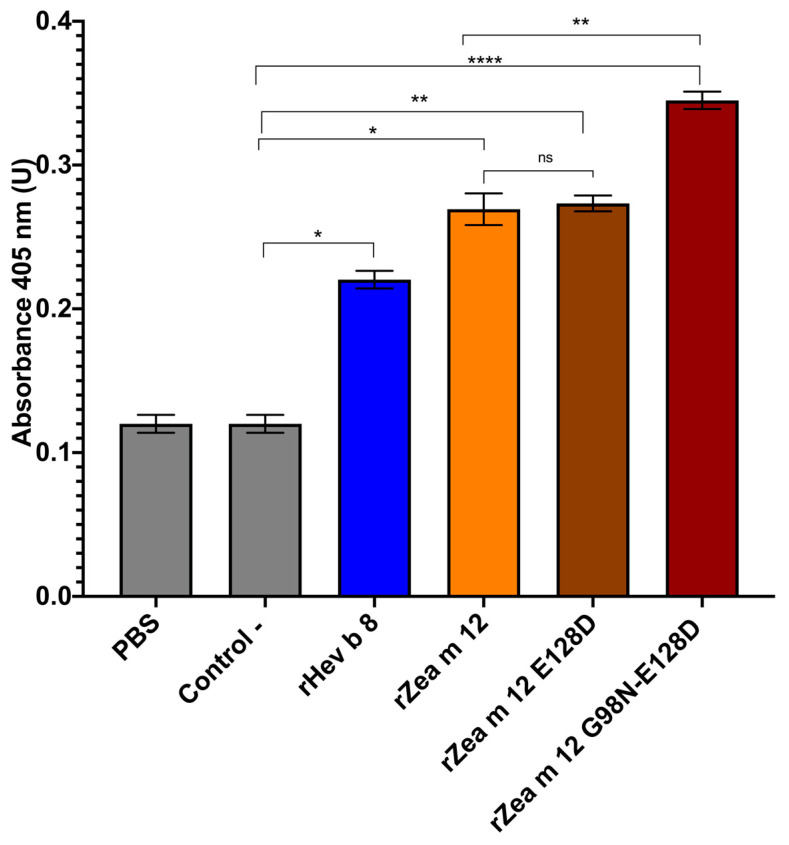
Specific IgE binding to rHev b 8, rZea m 12, and rZea m 12 mutants. The bars show IgE binding with the three profilins measured by indirect ELISA. The negative controls are two sera from non-allergic individuals and PBS as the background control. We used a pool of five individual sera for rZea m 12 and rZea m 12 mutants (1:5 dilution). The standard deviation of three different experiments is shown. The statistical analysis was performed by Sidak’s multiple comparisons tests (ns, not significant, *p* < 0.05 with one asterisk, *p* < 0.01 with two asterisks, and *p* < 0.0001 with four asterisks).

We performed indirect ELISA experiments with the rZea m 12 mutants (rZea m 12 E128D and rZea m 12 G98N-E128D) to identify if D128 and N98 are relevant in IgE binding with a pooled sera of profilin allergic patients that cross-reacted with Hev b 8. Notably, the double mutant rZea m 12 G98N-E128D showed higher IgE binding compared to rZea m a 12 and the controls (Figure 3), whereas the single mutant rZea m 12 E128D did not display any difference. This result demonstrates that N98 is essential for IgE binding in allergic patients.

### 3.4. Identification of Potential Cross-Reactive Residues in Plant Profilin Sequences by Mapping Recognition Sites of mAbs 1B4 and 2D10 (Anti-rHev b 8)

**Figure 4:**
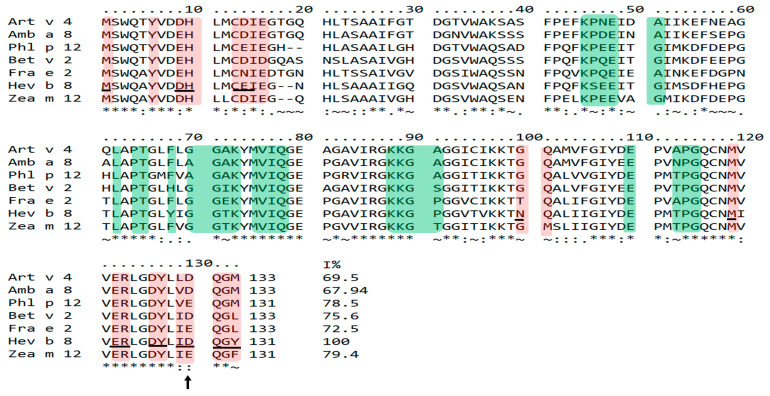
Mapping recognition sites of mAbs 2D10 and 1B4 in plant profilins’ sequences. The theoretical epitopes recognized by these two mAbs were previously determined by modeling their Fvs and docked with rHev b 8 [13]. Residues corresponding to mAb 2D10 recognition site are shaded in pink, while those conforming to the mAb 1B4 recognition site are shaded in green. The profilin sequences correspond to Amb a 8—*Ambrosia artemisiifolia* (ragweed), Phl p 12—*Phleum pratense* (Timothy grass), Bet v 2—*Betula verrucosa* (birch), Fra e 2—*Fraxinus excelsior* (ash), Hev b 8—*Hevea brasilensis* (latex), and Zea m 12—*Zea mays* (maize). The black arrow shows the D128 residue recognized by the mAb 2D10. This residue is also recognized by the 2F5/IgE on Hev b 8 (underlined in black), as reported by García-Ramírez et al. 2022 [12]. An asterisk (*) indicates fully conserved residues, a colon (**:**) indicates similar properties, a period (**.**) indicates weakly similar properties, and a tilde (**~**) indicates different properties.

We analyzed the profilins’ sequences to understand mAbs 1B4 and 2D10 binding towards plant profilins. We compared the theoretical epitopes of Hev b 8 recognized by 1B4 and 2D10 mAbs, reported by Mares-Mejia et al. 2020 [13] with the corresponding sequences in the profilins used in this study. Figure 4 shows the mAb 2D10 recognition sites shaded in pink and the 1B4 recognition sites in green. It is essential to mention that Hev b 8 has a D128. However, because the profilins of weeds (Art v 4 and Amb a 8) and trees (Bet v 2 and Fra e 2) have an insertion of two amino acids at position 17–20, in the sequence alignment, the D128 of Hev b 8 and other profilins is found in position 130. For this reason, we will refer to this position as 130 in all the profilins studied. The three profilins with poor binding with mAb 2D10 (Fra e 2, Zea m 12, and Phl p 12) have an E130, demonstrating that the D130 of Art v 4, Amb a 8, and Bet v 2 are relevant for mAb 2D10 recognition. The sequence alignment also shows that the conserved sequences constituting the recognition sites for 2D10 are in the amino and carboxyl-terminal regions. The mAb 1B4 recognition sites are shaded in green in Figure 4, where grass profilin (Phl p 12) has the highest number of identical residues with Hev b 8, even though the 1B4 recognition site includes one of the most variable parts (40–60). In contrast, the profilins Art v 4, Bet v 2, and Fra e 2, which had low recognition, showed more residue differences along the recognition site of 1B4. The relevant residue differences on profilins not recognized by 1B4 are E47 in Hev b 8 and Q47 in Fra e 2 and Bet v 2. Besides, residue T113 (Hev b 8) is A113 in Fra e 2 and Art v 4. This sequence alignment explains why mAbs 1B4 and 2D10 exhibit different binding patterns to plant profilins.

### 3.5. Identification of Relevant Structural Elements of Plant Profilins in mAb 2D10 (Anti-rHev b 8) Recognition

**Figure 5:**
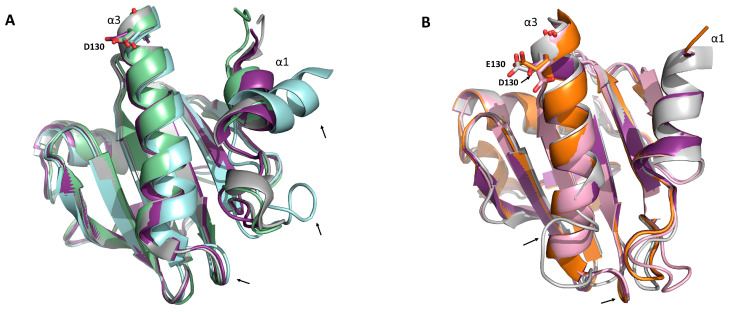
Structural comparisons of profilins which are highly and less recognized by the mAb 2D10. (**A**) Three-dimensional alignment of rHev b 8 (PDB 5FDS) (violet purple) shows the conserved D130 residue of Art v 4 (PDB 5EM0, gray), Amb a 8 (PDB 5EM1, green), and Bet v 2 (PDB 5NZB, cyan). The structural alignment of Hev b 8 with these three profilins corresponds to an RMSD of 0.7Å for Art v 4 and Amb a 8, and 0.9Å for Bet v 2. (**B**) Three-dimensional alignment of rHev b 8 (violet purple) that contains a D130 compared to the less-recognized profilins with a conserved E130. Fra e 2 is colored in pink (model obtained with AlphaFold), Zea m 12 (PDB 5FEF, orange), Phl p 12 (PDB 7KYW, gray). The RMSD of Zea m 12 compared with Hev b 8 is 0.3 Å, for Phl p 12 is 0.5 Å, and 0.4 Å for Fra e 2. The black arrows highlight the profilins’ structural differences.

To determine relevant residues that could explain 2D10 recognition, we performed structural alignment analysis with Hev b 8 and the other reported structures for each profilin. Figure 5 shows the three-dimensional structure alignment of Art v 4, Amb a 8, Bet v 2, and Hev b 8 that have D130 in the α-helix 3. Figure 5B shows those less recognized by this mAb: Fra e 2, Zea m 12, and Phl p 12 superimposed with Hev b 8 that has E130. The two structural differences of Bet v 2 are a deviated α-helix 1, and therefore the loop that connects to the β-strand 1 is also moved to the right (Figure 5A). The α-helix 3 in Amb a 8, Ar v 4, Zea m 12, Fra e 2, and Bet v 2 has a similar length and orientation to the one present in Hev b 8 (violet purple), with a short loop that connects it with β-strand 7. Conversely, the Phl p 12 (grey) α-helix 3 is shorter, with a longer loop comprising residues 106–116 (Figure 5B). These structural differences might be relevant for interacting these profilins with 2D10.

### 3.6. Electrostatic Potential Analysis of Plant Profilins

**Figure 6:**
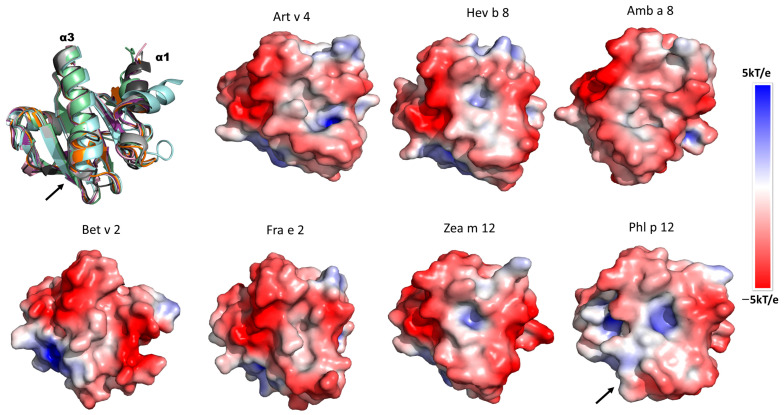
Comparison of the electrostatic potential surfaces in plant profilins’ allergens. Three-dimensional alignment shows the orientation of electrostatic potential charge in profilins’ surfaces on α-helices 1 and 3. The profilins are ordered from the highest to the lowest recognition by 2D10. The positive charge patches are in blue, and the negative in red. The black arrows highlight structural differences in Phl p 12 from α-helix 3 to the loop that connects β-strand 7.

To identify charges on the profilins’ surfaces that could be relevant for mAb recognition, we evaluated the electrostatic potential of the different profilins. They are ordered from the highest to the lowest binding with mAb 2D10 in Figure 6. We observe a conserved negative charge along α-helices 1 and 3 surfaces in most profilins, with Phl p 12 exception. On Phl p 12, the negative charge surface decreases, and in the loop that connects α-helix 1 with β-strand 7 (Figure 5B) (residues 106–116 in Figure 4), there are no negative patches, which explains why Phl p 12 was not recognized by 2D10. It is worth noting, however, that the structure of Phl p 12 used here is an example of dimeric profilin where α-helix 3 unfolded to generate two disulfide bonds between the molecules in the dimer [16]. Monomeric Phl p 12 may have similar properties to the other profilins. Another relevant difference between Art v 4 with Hev b 8 is the number of negative charges, which increases in the extra α-helix 1 region of Art v 4 (Figure 5A) (residues 14–18 in Figure 4), which explains mAb 2D10’s higher recognition for Art v 4 compared to Hev b 8.

## 4. Discussion

Here, we evaluated the IgG mAbs 1B4 and 2D10 recognition towards several plants profilins, including profilins from trees (rBet v 2.0101 and rFra e 2.2), weeds (rAmb a 8.0101 and rArt v 4.0101), grass (rPhl p 12.0101), and maize (rZea m 12.0105), to identify relevant amino acids involved in mAbs recognition, and compare them with the reported IgE epitopes on rHev b 8.0102 to target residues involved in IgE cross-reactivity that could be blocked with these antibodies.

The first relevant result was the different binding patterns of anti-rHev b 8 mAbs 1B4 and 2D10 towards allergenic plant profilins with amino acid sequence identities of 67 to 79%. mAb 2D10 strongly recognized weeds’ profilins (rArt v 4 and rAmb a 8), latex profilin rHev b 8, and to a lesser extent, profilins from trees (rBet v 2 and rFra e 2). Conversely, 1B4 recognized the profilins ordered from the highest to the lowest recognition: rHev b 8, maize (rZea m 12), grass (rPhl p 12), and weed (rAmb a 8) (Figure 1). We also demonstrated that this mAb detected tomato profilin (rSol l 1) [13]. Therefore, the high capacity of mAbs to cross-react and recognize profilins from distinct sources can be used to detect and quantify profilins from natural sources and for diagnostic purposes [9,10,20].

The second and most significant result was that the IgE from pooled sera from latex- and maize-allergic patients showed higher binding with the double mutant (G98N-E128D) (Figure 3), compared to the single mutant rZea m 12 E128D. This result agrees with our previous findings, where murine 2F5/IgE showed 56% recognition with the rZea m 12 double mutant [12]. We must emphasize that we used a pool of sera from allergic patients; in addition, the IgE antibodies of individuals are polyclonal and have a different epitope recognition repertoire [21,22]. Therefore, 2F5/IgE shares similar epitopes, including N98, recognized by IgE antibodies from latex- and maize-allergic patients. Besides, various researchers have established that the major profilin IgE epitope is located on α-helices 1 and 3 [23,24,25,26,27]. This demonstrated that N98 is a relevant residue for IgE recognition.

Another significant result was that 2D10 showed increased binding for the mutant rZea m 12 E128D (Figure 2). Therefore, D128 at the carboxyl-terminal α-helix 3 of profilins (Figure 5A), which we have demonstrated to be involved in IgE/2F5-binding to latex profilin (rHev b 8), is implicated in IgE cross-reactivity [12]. Additionally, these mAbs inhibited polyclonal antibodies (from allergic patients’ sera), 23% for 1B4 and 74% for 2D10. These results suggest that 2D10 recognized a similar conformational epitope on rHev b 8 [13]. The recognition sites on the rZea 12 mutants with 1B4 allowed us to demonstrate that it does not share the recognition site with 2D10, carboxyl-terminal α-helix 3. This result corroborates the theoretical recognition site established by Mares-Mejia et al. (2020) [13]. Therefore, in the present work, we suggest that D130 from the α-helix 3 is relevant for mAb 2D10 recognition.

Furthermore, the sequence alignment analysis of the plant profilins suggests that the conserved D130 of rArt v 4, rAmb a 8, and rBet v 2 (D128 in Hev b 8) is essential for 2D10 recognition. In line with this, the profilins containing an E130 (rPhl p 12, rFra e 2, rZea m 12) show less binding (Figure 4). The latter could be explained by the longer side chain of residue E130, which generates a steric hindrance (Figure 5B). These observations demonstrate that D130 and E130 of profilins greatly influence their binding to mAb 2D10. Based on our previous findings with Fab 2F5/IgE, residue D128 establishes two salt bridges with residues R50 and R52 (CDR2-H) of the paratope, and residue N98 interacts through hydrogen bonds with T55 in the CDR2-H stabilizing the complex [12]. Other authors have reported that E128 is implicated in IgE recognition for rCuc m 2 using synthetic peptides spanning the Cuc m 2 binding surface with IgE from profilin melon-allergic patients [2,11].

Moreover, based on the molecular docking of both mAbs 1B4 and 2D10 of the Fvs-rHev b 8 complexes [13], we identified that the 2D10 recognition site corresponds to one of the most conserved regions in profilins (residues 1–16 and 119–133 in Figure 4), that are the N- and C- terminal α-helices 1 and 3; which is a common binding site for the poly(L-Pro) ligand [27]. The recognition site for 1B4 is in the most variable part of the sequence of the plant profilins studied (residues 40–70 in Figure 4). Relevant residue differences on these profilins are E47 in Hev b 8 and Q47 in Fra e 2 and Bet v 2, which are not recognized by 1B4. In addition, residue T113 (Hev b 8) is A113 in Fra e 2 and Art v 4. Therefore, mAb 1B4 recognized rZea m 12, rPhl p 12, and rAmb a 8 that exhibit more conserved residues with Hev b 8.

Here, we also found that rAmb a 8 was highly recognized by 2D10 (88% relative to binding to rHev b 8) and was recognized by 1B4 (56%). Likewise, rArt v 4 exhibited higher recognition to 2D10 compared to rHev b 8, and rPhl p 12 was recognized (72%) by 1B4. Conversely, two profilins, rBet v 2 and rFra e 2, which were less recognized by 2D10 (54% and 42%, respectively), might have a low ability to inhibit IgE binding. We must highlight that the mixture of these two mAbs (1B4 and 2D10) blocks the recognition of IgE and IgG4 antibodies from sera of latex-allergic and maize-allergic patients by 90% and 40%, respectively, which revealed that these mAbs have overlapping recognition sites with the specific-profilin antibodies from sera of allergic patients [13]. Additionally, the mAbs 1B4 and 2D10 (anti-rHev b 8) showed a unique set of profilin-binding patterns, such as in studies with IgE from latex-allergic patients [28]. Immunoblot inhibition experiments demonstrated those results with IgE from sera of latex-allergic patients using mugwort (*Artemisia vulgaris*), ragweed (*Ambrosia elatior*), Timothy grass (*Phleum pratense*) extracts, and rBet v 2 (*Betula verrucosa*), which revealed that profilins from weeds and grass share common epitopes with latex, but not with Bet v 2 [28]. These data allow us to hypothesize that our two antibodies could have an IgE inhibitory effect with rAmb a 8, rArt v 4, and rPhl p 12; therefore, we should undertake future studies on IgE-inhibition experiments to prove this.

The structural alignment of plant profilins indicates that Amb a 8, Art v 4, Zea m 12, Bet v 2, and Fra e 2 have a similar length in the α-helices 1 and 3 with Hev b 8 (Figure 5), which explains 2D10 recognition (Figure 1). Due to its conformation as a dimer, rPhl p 12 has the most relevant structural difference, in which the α-helix 3 is short and consequently has a longer loop that connects with β-strand 7. In comparison, the other profilins have a shorter loop [16]. Therefore, the rPhl p 12 conformational epitopes differ along the α-helix 3 with the rest of the plant profilins. This structural difference may explain why rPhl p 12 was not recognized by 2D10. Furthermore, Bet v 2 has a clear structural difference in α-helix 1, caused by an internal C13–C115 disulfide bond [29] that may contribute to a different arrangement of antigenic determinants that could explain its lower binding (54%), as compared to the mAb binding towards rHev b 8 (Figure 1). Westritschnig et al. designed a restructured hypoallergenic variant of *Timothy grass* profilin (Phl p 12-rs) “tail-to-head”, meaning that the C terminus of Phl p 12 was at its N terminus and the N terminus was at its C terminus [30]. This implies that the conformational epitopes in α-helices 1 and 3 of Phl p 12-rs were severely compromised. Consequently, Phl p 12-rs showed a notably reduced IgE reactivity and allergenicity [30]. These results confirmed our conclusion that the structural differences at α-helices 1 and 3 in plant profilins change the shape of the conformational epitope and impact antibody recognition.

The electrostatic potential analysis indicates that the distribution of negative charge patches on the profilins’ surface at the α-helices 1 and 3 are relevant for the 2D10 recognition. These negative charge patches on profilins are suitable for their interaction with the positive amino acid of the paratope [16]. Structural studies of Fab 2F5/IgE-rHev b 8 complexes demonstrated that D128 from the epitope interacts with two basic residues, R50 and R52 of H-CDR2 of the paratope [12]. Additionally, structural analysis of nine murine Fab IgG-allergen complexes also demonstrated that residues D and E are frequently found in epitopes on allergen surfaces [31]. For example, in the Api m 2-Fab 21E11 complex crystal structure, residue D146 of the epitope interacted with the Y32 of the L-CDR 1 (paratope), and D145 interacted with the G93 of the L-CDR 3 paratope through hydrogen bonds [32]. Another example is in the Bet v 1-Fab BV16 complex, in which E45 of the epitope interacts with W104 H-CDR 1 and W33 H-CDR 3 through two short hydrogen bonds [33].

In summary, the mAbs binding to different plant profilins and their broad cross-reactivity may represent a promising tool in allergy for both diagnoses [9,10,20] and epitope mapping [32,33,34]. Humanized mAbs with similar characteristics to mAb 2D10 may have therapeutic implications in personalized therapies [35,36,37,38].

## 5. Conclusions

This study has demonstrated that the mAb 2D10 tightly bound the weed profilins rArt v 4, rAmb a 8, and to a lesser extent rBet v 2, rFra e 2, and rZea m 12, while mAb 1B4 showed higher recognition to rPhl p 12 and rZea m 12, and less binding for rAmb a 8. The rZea m 12 mutant E128D demonstrated that residue D130 in plant profilins is essential for mAb 2D10 recognition. On the contrary, 1B4 did not show any recognition difference with rZea 12 mutants, demonstrating that it does not share the recognition site with 2D10. Indeed, sequence and structural alignment showed that D130 in α-helix 3 of Amb a 8, Art v 4 y Bet v 2 is relevant to 2D10 recognition. In contrast, the profilins containing E130 (rPhl p 12, rFra e 2, and rZea m 12) have less binding. The conformational differences and the distribution of negative patches on the profilins’ surface in α-helices 1 and 3 contribute to 2D10 recognition, which may be relevant to explain their IgE cross-reactivity. Altogether, these findings suggest that the development of humanized mAbs with similar characteristics to mAb 2D10 could have therapeutic implications in personalized therapies.

## Data Availability

Not applicable.
